# Fetal programming of adrenal PNMT and hypertension by glucocorticoids in WKY rats is dose and sex-dependent

**DOI:** 10.1371/journal.pone.0221719

**Published:** 2019-09-04

**Authors:** Sandhya Khurana, Julie Grandbois, Sujeenthar Tharmalingam, Alyssa Murray, Kelly Graff, Phong Nguyen, T. C. Tai

**Affiliations:** 1 Medical Sciences Division, Northern Ontario School of Medicine, Sudbury, Ontario, Canada; 2 Department of Biology, Laurentian University, Sudbury, Ontario, Canada; 3 Department of Chemistry and Biochemistry, Laurentian University, Sudbury, Ontario, Canada; 4 Biomolecular Sciences Program, Laurentian University, Sudbury, Ontario, Canada; Max Delbruck Centrum fur Molekulare Medizin Berlin Buch, GERMANY

## Abstract

Biochemical changes *in utero* may alter normal fetal development, resulting in disease later in life, a phenomenon known as fetal programming. Recent epidemiological studies link fetal programming to negative health outcomes, such as low birth weight and hypertension in adulthood. Here, we used a WKY rat model and studied the molecular changes triggered by prenatal glucocorticoid (GC) exposure on the development of hypertension, and on the regulation of phenylethanolamine N-methyl transferase (PNMT), the enzyme responsible for biosynthesis of epinephrine, and a candidate gene linked to hypertension. Clinically, high doses of the synthetic GC dexamethasone (DEX) are used to treat infant respiratory distress syndrome. Elevated maternal GCs have been correlated with fetal programming of hypertension. The aim of this study was to determine if lower doses of DEX would not lead to detrimental fetal programming effects such as hypertension. Our data suggests that prenatal stress programs for increased expression of PNMT and altered regulation of PNMT in males and females. Importantly, we identified that DEX mediated programming was more apparent in the male rats, and the lower dose 10μg/kg/day of DEX did not lead to changes in blood pressure (BP) in female rats suggesting that this dose is below the threshold for programming of hypertension. Furthermore, sex-specific differences were observed in regards to programming mechanisms that may account for hypertension in males.

## Introduction

Emerging evidence suggests that *in-utero* insults experienced during critical stages of fetal development lead to an unfavourable biochemical environment causing compensatory fetal adaptations. These adaptive mechanisms occur via alterations in structure and/or function of organs that may be permanent, leading to an increased risk of developing disease later in life, a phenomenon known as fetal programming of adult disease [1]. Maternal undernutrition, hypoxia, exposure to alcohol, and high levels of glucocorticoids are examples of stressors that can impact fetal development, resulting in fetal programming. Exposure to prenatal stress has been strongly associated with the development of the “thrifty phenotype”, leading to increased risk of cardiovascular disease, kidney disease, metabolic syndrome and hypertension amongst others, exemplifying the crucial role of early development in-utero in determining the onset of disease [2,3].

Numerous researchers have identified that stress hormones such as glucocorticoids (GCs) contribute to fetal programming of hypertension; however, the molecular mechanisms are not fully understood [4,5]. GCs are hormones that are lipophilic molecules that easily cross the placenta, and can induce a stress-like state upon the developing fetus [2]. Placental GC concentrations are substantially low in comparison to maternal levels due to 11β-dehydroxysteroid dehydrogenase 2 (11β-HSD2), which converts bioactive GCs into their inactive form [2]. However, synthetic GCs such as dexamethasone (DEX) are weak substrates for 11β-HSD2, and are able to cross the placenta and exert their effects onto the fetus [6].

Exposure of the fetus to GCs may occur endogenously (maternal stress) or exogenously (to treat neonatal respiratory distress syndrome). Clinically, DEX is administered to pregnant women at risk for preterm birth to promote fetal lung maturation. In humans, and animal studies, exposure to DEX and other GCs during pregnancy has been linked to intrauterine growth impairment, development of hypertension and cardiovascular diseases (CVD) in adulthood [1,5,7]. Although the mechanisms are not completely understood, altered expression of receptors, enzymes, transporters, and ion channels leads to deviations in organ development, and collectively these physiological changes result in hypertension, diabetes, and CVD [4].

PNMT is the terminal enzyme in the catecholamine biosynthetic pathway, converting norepinephrine to epinephrine. Genetic mapping and microarray studies have linked PNMT to hypertension, making it a candidate gene for hypertension [8,9]. We, and others, have shown that the expression of PNMT is higher in spontaneously hypertensive rats (SHRs), compared to their normotensive counterparts, the Wistar Kyoto (WKY) rats; however no polymorphisms were detected in the PNMT gene which could account for the overexpression [9–11]. In humans, genotyping studies identified polymorphisms in the PNMT gene promoter that correlated with the development of hypertension in African-Americans, but not in white Americans or in individuals of European descent suggesting that other mechanisms could account for overexpression of PNMT [12,13].

The PNMT gene promoter is regulated by stress sensitive transcription factors including early growth response-1 (Egr-1), activating enhancer binding protein 2 (AP-2), specificity protein 1 (Sp1), and glucocorticoid receptor (GR) [14–17]. Hormonal regulation is achieved via the hypothalamic-pituitary-adrenal (HPA) axis; GCs secreted under stress bind to GR. The GC-GR complex then binds to GC response elements (GRE) on the PNMT promoter [16,18]. The developmental factor AP-2 requires the presence of GCs for significant activation of the PNMT promoter [19,20]. Neural regulation of the PNMT promoter is triggered through the sympatho-adrenal (SA) axis, where the splanchnic nerve releases acetylcholine and pituitary adenylate cyclase-activating polypeptide (PACAP), activating Egr-1 and Sp1 in adrenal chromaffin cells via cAMP/PKA, PKC and MAPK signalling pathways [21,22]. All 4 transcription factors can independently activate PNMT transcription, and/or synergistically amplify PNMT promoter activity beyond their individual effects [16,18].

Recent evidence supports that overexpression of the PNMT gene in adrenal glands, heart and brainstem of SHRs is caused by altered transcriptional regulation, and contributes to their hypertensive phenotype [11,23,24]. Studies from our lab have also previously demonstrated that the expression of adrenal PNMT is elevated when offspring are exposed to high doses of DEX prenatally, caused by altered transcriptional mechanisms [25]. A lower prenatal DEX dose that does not cause elevated BP in rat offspring could show that a lower, safer alternative might exist in humans. The objective of this study was to identify doses of prenatal DEX that do not result in fetal programming of hypertension, and the dysregulation of catecholamine biosynthetic enzymes, and other stress sensitive genes.

## Methods

### Animal protocols and housing

The study was carried out in accordance with guidelines from the Canadian Council on Animal Care. The protocol was approved by the Animal Research Laurentian University Animal Care Committee (AUP6013736). Food and water were available *ad libitum*. Rats were kept in a 12-hour light-dark cycle, with the light phase being 6:00 am to 6:00 pm.

WKY male and female rats (aged 8 weeks; Charles Rivers Laboratory; Montreal, QC, CA) were acclimatized for 2 weeks. One male was introduced to three female rats for five days. Females were monitored daily for vaginal plugs, and subsequently were housed individually. Pregnant females were administered subcutaneous injections of DEX (prepared in 0.9% NaCl, 4% ethanol) at concentrations of 10, 50 or 100 μg/Kg/day (denoted as 10-DEX, 50-DEX, or 100-DEX, respectively), or a vehicle solution throughout the third trimester; days 15–21 [26]. The naïve group did not receive any injections. Pups were weaned at 3 weeks of age, separated by sex, and housed 2–3 rats per cage. For all groups, a minimum of n = 3 mothers were used; n = 6–12 animals were analyzed per sex, per group with care to take at least 2 animals per sex, per litter for analyses. In cases where this wasn’t possible (for example only 1 male in the litter) remainder pups were randomly chosen from other litters for making up n values.

### Physiological measurements

Offspring were weighed at 5, 10, and 14 days of age, and subsequently once a week. At three weeks of age, offspring were acclimated to a non-invasive tail-cuff plethysmography (CODA 6, Kent Scientific, Torrington, CT, USA); BP was monitored 3-times /week from 4–19 weeks of age [11,25,27].

### Tissue collection and qRT-PCR

At 19 weeks, rats were anaesthetized by an intraperitoneal administration of 75 mg of ketamine (Ketalean, CDMV Inc., Montreal, QC, CA) and 5 mg xylazine (Sigma, St. Louis, MO, USA) per Kg of body weight. Animals were sacrificed and trunk blood collected in Vacutainer vials (BD, Franklin lakes, NJ, USA). Plasma aliquots were stored at -80°C until further processing. Adrenal glands were immediately frozen on dry ice and stored at -80°C until further processing. RNA extraction and qRT-PCRs were performed as previously described, and primers for PNMT, TH, DBH, PNMT and the transcription factors were as previous [24,25]. Primers for PAH, SLC9A3 and CYP2E1 were designed as follows: PAH: F: 5’-GCTGCTAAGCTAGACACCTCA-3’, R: 5’-CTTGTTTCCTGCCCAAAGTCT-3’; SLC9A3 F: 5’-CCGCCTCAGCAACAAATCAG-3’, R: 5’-GAGCCTGTATCACATGTGTGTGG-3’, CYP2E1 F: 5’TTCACCAAGTTGGCAAAGCG-3’, R: 5’- CCTTGACAGCCTTGTAGCCA-3’.

### ELISA

Epinephrine (CAT ELISA, Rocky Mountain Diagnostics, CO, USA), and Corticosterone (Corticosterone ELISA kit, R&D Diagnostics, MN, USA) were measured in the plasma as per the manufacturer’s instructions.

### Statistical analyses

The physiological measurements are presented as mean ± SEM with individual pups being used as a unit of comparison. Fold-changes in gene expression were calculated by relative quantification (ΔΔC_t_) of qRT-PCR threshold cycles (C_t_) as per Livak and Schmittgen using Ct values of housekeeping gene, GAPDH, and is presented as mean ± SD [28]. Statistical significance between groups was determined by ANOVA followed by Dunnetts or Tukey’s post-hoc test (GraphPad Prism, La Jolla, CA, USA). Results with p ≤ 0.05 were considered statistically significant.

## Results

### Physiological measurements

**Fig 1:**
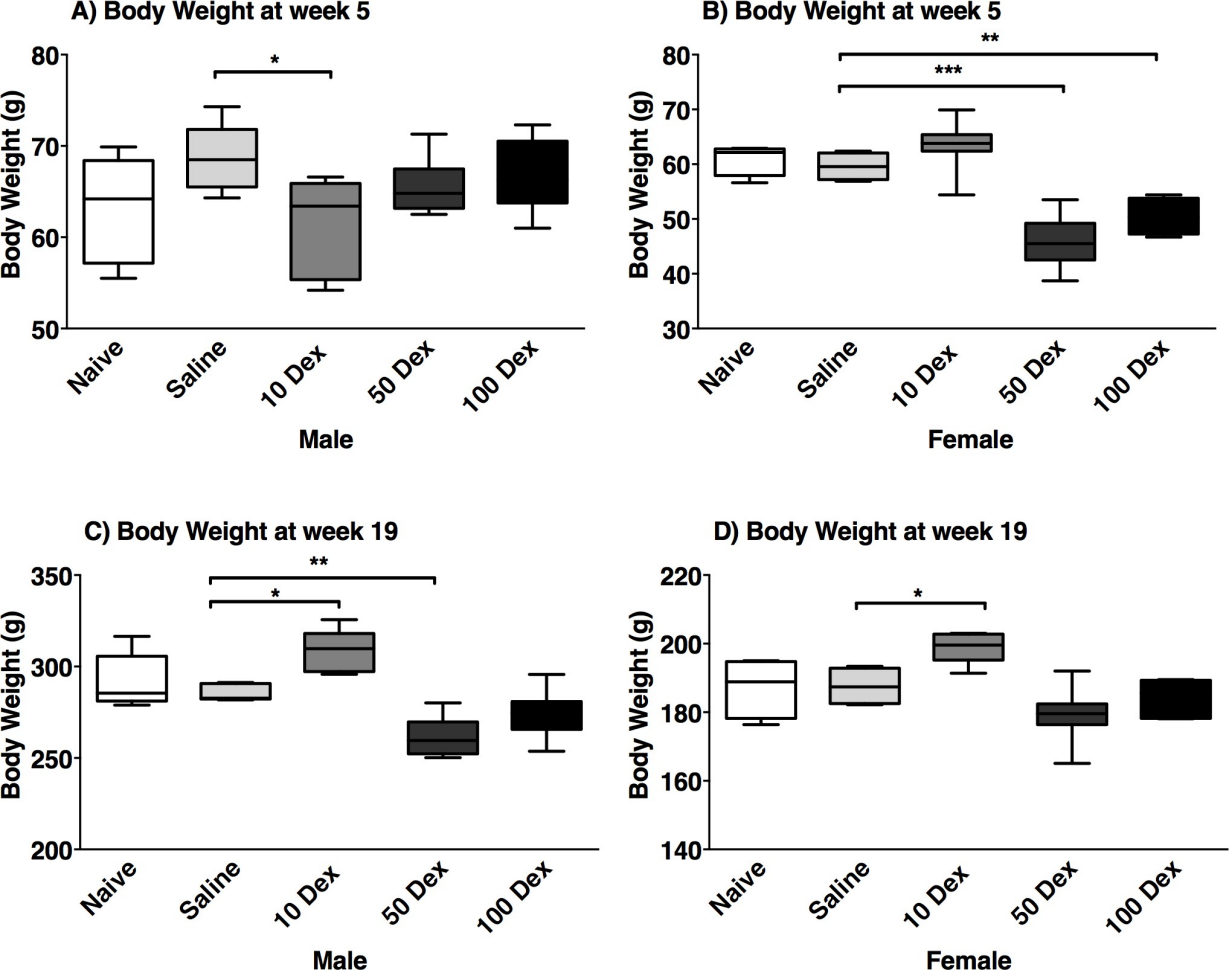
Body weight of DEX exposed offspring. Body weight (grams; g) of male (left) and female (right) offspring at 5 weeks (A, B), and 19 weeks of age (C, D), of naive, saline, 10-DEX, 50-DEX, and 100-DEX groups. Data are displayed as a box plot with average, minimal, and maximal weights ± SEM. ANOVA: Statistical significance between naive and DEX-treated groups is shown by: * p < 0.05, ** p < 0.01, and *** p < 0.001.

Body weight was measured in the offspring of naïve, saline, and prenatally DEX-exposed animals (Fig 1). In both sexes, no statistical differences were detected between the saline and the naive groups, at any age. At 5 weeks of age, the average body weight of prenatally DEX-exposed animals was lower than either naïve or saline counterparts, in all doses in the males. In the females, the 10-DEX dose was least affected and weights were comparable to the naïve and saline groups. By 19 weeks, the growth of the animals in both sexes showed higher weight compared to saline in the 10-DEX group, while with the 50-DEX and 100-DEX groups, animals weighed almost as much as controls, possibly due to catch-up growth in the latter.

**Fig 2:**
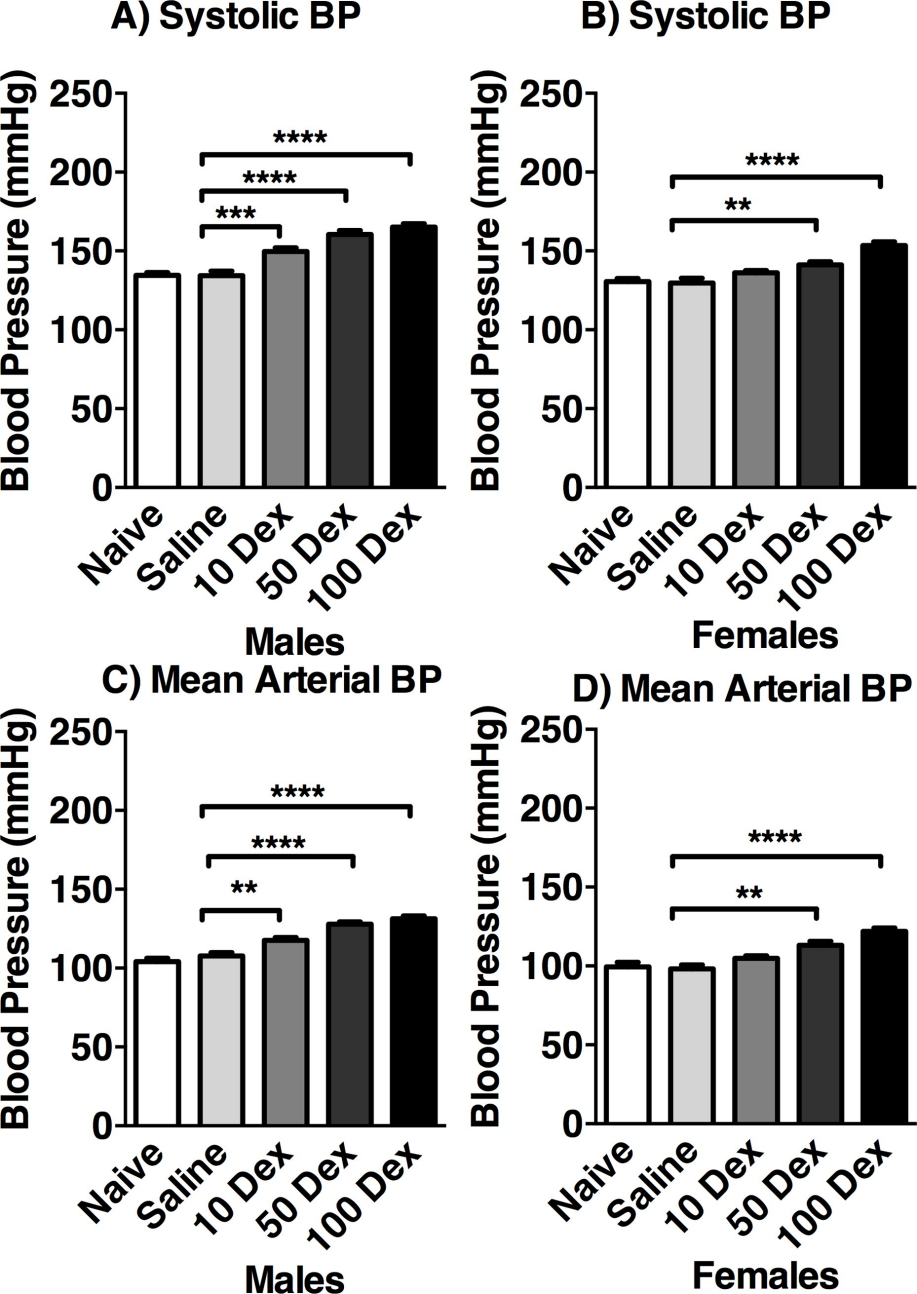
Blood pressure of 19-week-old DEX exposed offspring. Systolic (A, B) and mean arterial blood pressure (C, D) of male (left) and female (right) naive, saline, 10-DEX, 50-DEX, and 100-DEX offspring are expressed in mmHg. Data are expressed in mean ± SEM. ANOVA: Statistical significance between naive and DEX-treated groups is shown by: * p < 0.05, ** p < 0.01, *** p < 0.001, and **** p < 0.0001.

BP was measured from 4–19 weeks of age. For both sexes, no significant differences were observed between the naïve and saline groups. At 19 weeks (Fig 2), male offspring showed increased systolic BP in 10-DEX (149.5 ± 2.35 mmHg), 50-DEX (160.4 ± 2.46 mmHg), and 100-DEX (165.1 ± 2.04 mmHg) groups, compared to naïve (134.5 ± 1.86 mmHg), and saline (134.43 ± 2.77 mmHg). In females, systolic BP was higher in 50-DEX (141.2 ± 1.87 mmHg), and 100-DEX (17%; 153.4 ± 2.32 mmHg), compared to naïve (130 ± 1.97 mmHg) and saline controls (128.1 ± 2.88 mmHg). Similar results were found for mean arterial pressure for both sexes.

### Adrenal PNMT and transcriptional regulators

**Fig 3:**
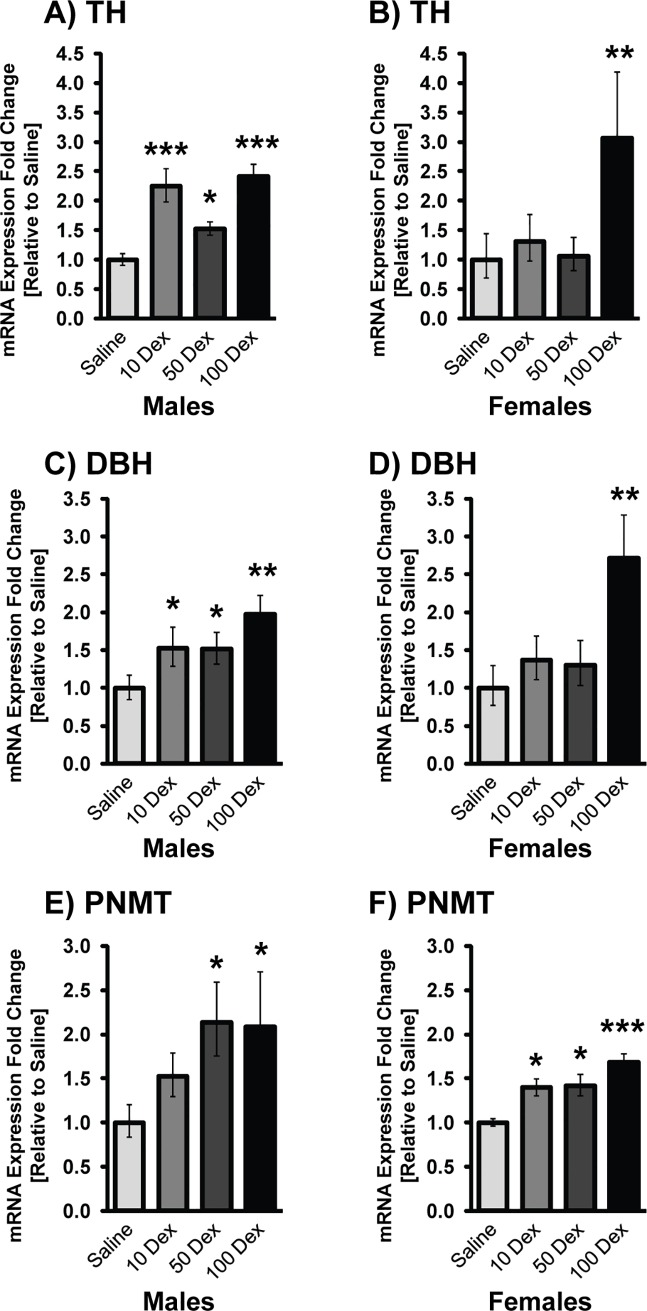
Gene expression of catecholamine biosynthetic enzymes in adrenal glands of 19-week-old prenatally DEX-exposed offspring. mRNA levels of TH (A, B), DBH (C, D), and PNMT (E, F) in male (left) and female (right) offspring of saline, 10-DEX, 50-DEX, and 100-DEX treatment groups. Data is expressed as mean fold change ± SEM. ANOVA: Statistical significance between saline and DEX-treated groups is shown by: * p < 0.05, ** p < 0.01, and *** p < 0.001.

Transcripts of TH, DBH, and PNMT were analyzed by qRT-PCR in the adrenal glands of DEX-treated animals (Fig 3). Since the body weight and BPs of the naïve and saline groups were indistinguishable from each other, all further comparisons of treatment groups were made to the saline. Predominantly, a dose response was seen in transcripts of TH (10-DEX: 2.25 fold; 50-DEX: 1.53 fold and 100-DEX: 2.41 fold), DBH (10-DEX: 1.53 fold; 50-DEX: 1.51 fold and 100-DEX: 1.91 fold) and PNMT (50-DEX: 2.13 fold and 100-DEX: 2.09 fold) in the males compared to saline. Similarly, females also showed changes in TH (100-DEX: 3.06 fold), DBH (100-DEX: 2.72 fold), and PNMT (10-DEX: 1.40 fold; 50-DEX: 1.42 fold and 100-DEX: 1.68 fold). In the females, the majority of significant changes were seen in the higher doses of DEX exposure, while the lower doses were comparable to the saline group.

**Fig 4:**
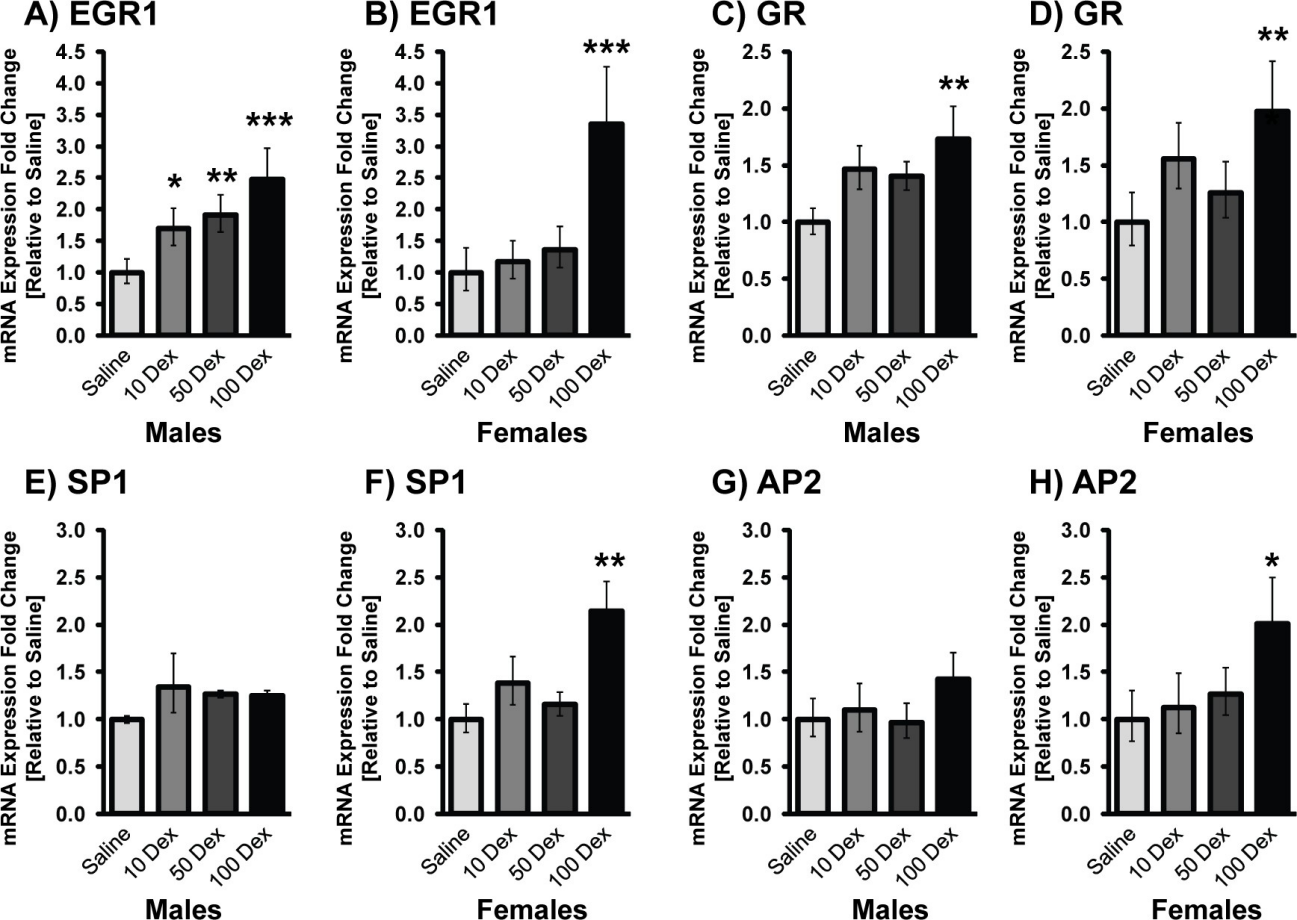
mRNA levels of PNMT transcriptional regulators in adrenal glands of 19-week-old prenatally DEX-exposed offspring. mRNA levels of Egr-1 (A,B), GR (C,D), Sp1 (E, F), and AP-2 (G, H) in male (left) and female (right) offspring of saline, 10-DEX, 50-DEX, and 100-DEX treatment groups. Data is expressed as mean fold change ± SD. ANOVA: Statistical significance between saline and DEX-treated groups is shown by *, p < 0.05, ** p < 0.01, and *** p < 0.001.

To verify if altered PNMT was a consequence of changes in the mRNA levels of its key transcriptional regulatory factors, expression of Egr-1, AP-2, Sp1, and GR were determined (Fig 4). Overall, in both sexes a clear dose response was observed with minimal changes in the 10-DEX group, and significantly higher alterations observed in the 100-DEX group. In the males, changes in Sp1 and AP2 were not significant, even in the 100-DEX group, while Egr-1 and GR were elevated 2.47 fold and 1.73 fold respectively. Interestingly, Egr-1 was upregulated even at the lowest DEX dose suggesting lower stress sensitivity for the activation of this transcription factor in males. In the females, all 4-transcription factors were significantly elevated compared to the saline group at 100-DEX (Egr-1: 3.35 fold; GR: 1.97 fold; Sp1: 2.14 fold, and AP-2: 2.01 fold). Although some of these transcription factors were elevated at the lower doses (10DEX), PNMT transcript was not increased, likely because promoter activation requires cooperative interaction of all factors as previously shown [20].

### Plasma corticosterone and epinephrine

**Fig 5:**
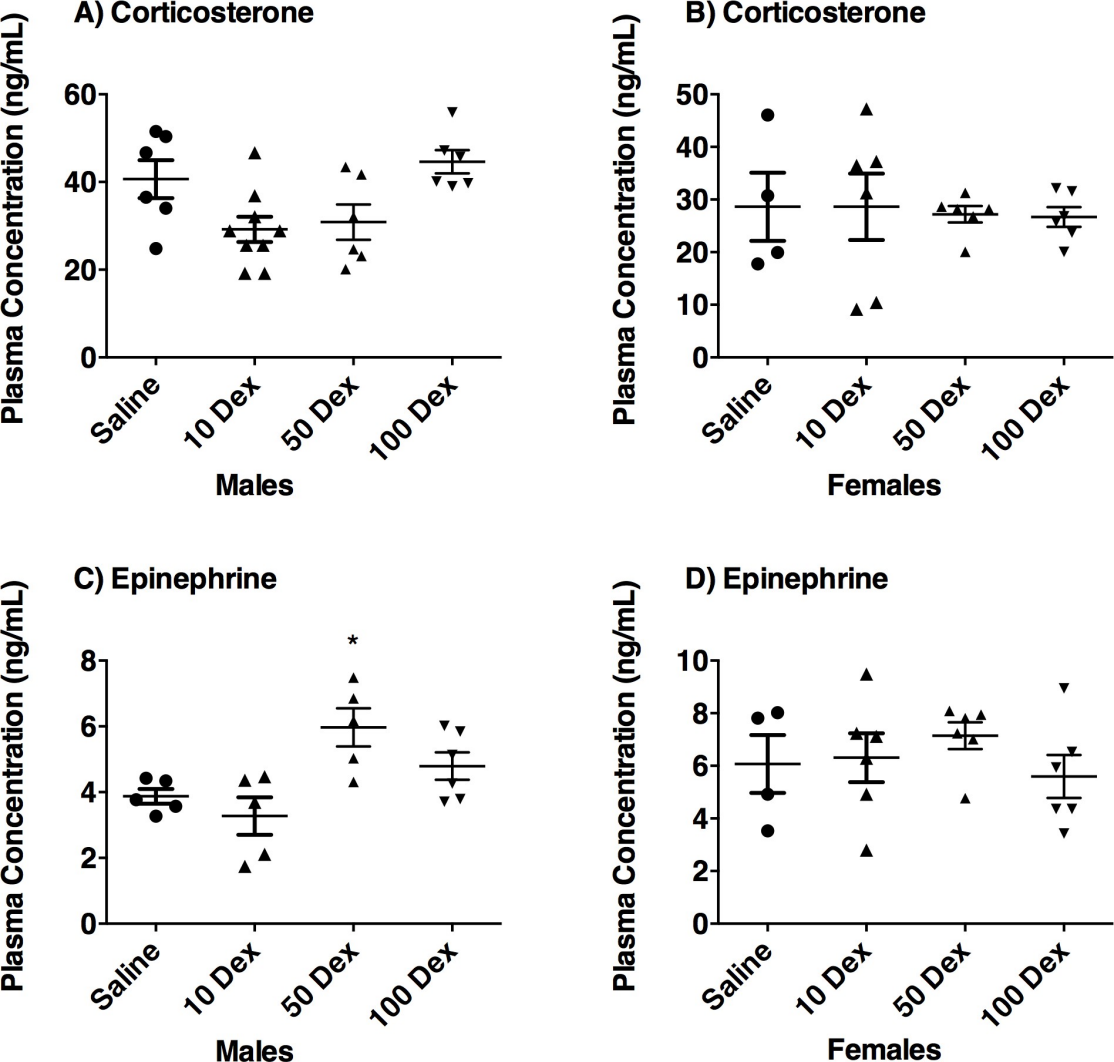
Effect of prenatal DEX exposure on plasma corticosterone and epinephrine levels. Plasma corticosterone (A, B), and epinephrine levels (C, D), in male and female offspring from saline or DEX-exposed animals. Fold changes between saline controls versus DEX-treated animals are presented as mean ± SEM. Significant difference between groups designated as *, p ≤ 0.05.

Plasma corticosterone and epinephrine were quantified by ELISA (Fig 5). Corticosterone levels remained unchanged in all three DEX-doses regardless of sex. Epinephrine showed trends towards increased levels in the 50 and 100-DEX males, but no changes were observed in the females.

### Adrenal stress-sensitive genes

**Fig 6:**
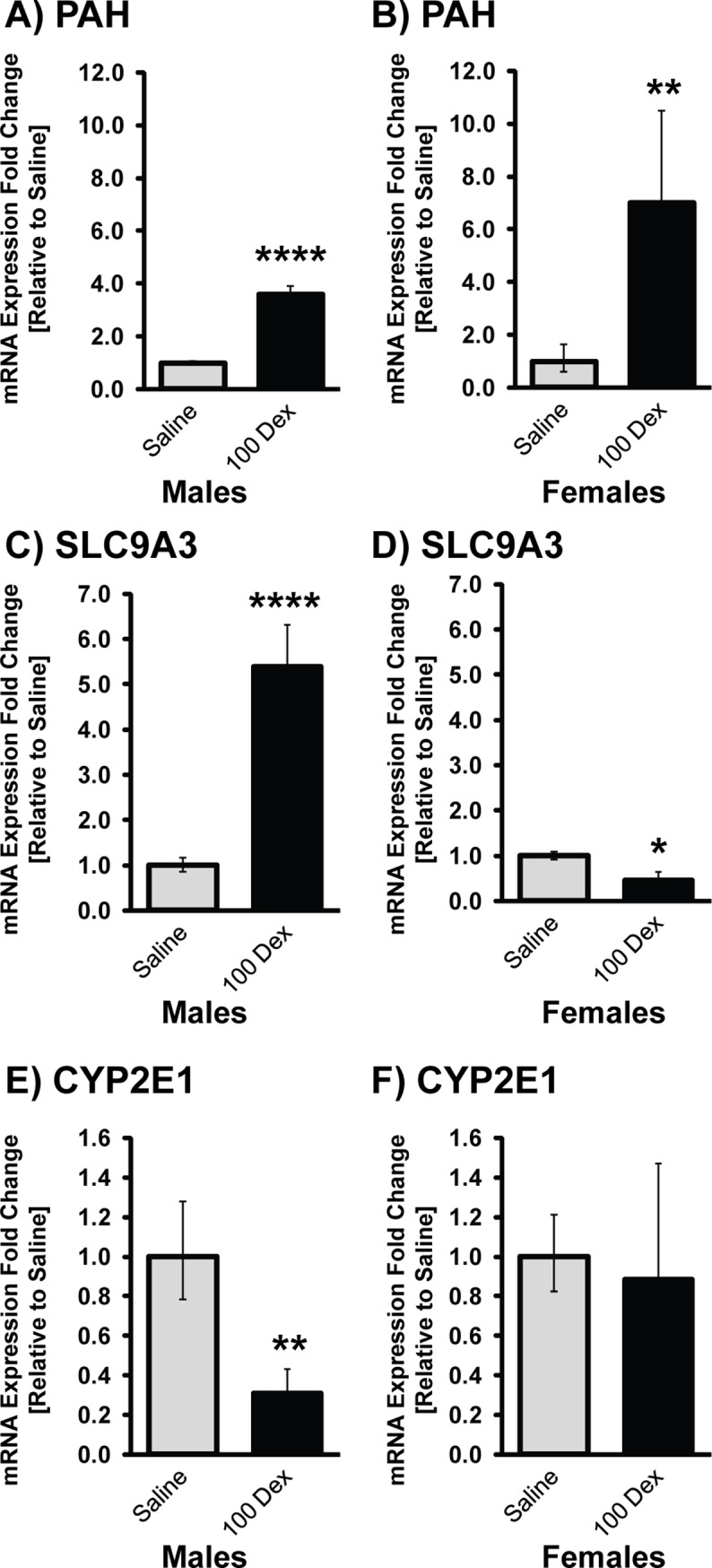
mRNA levels of stress related genes in adrenal glands of 19 week old prenatally DEX-exposed offspring. mRNA levels of PAH (A,B), SLC9A3 (C,D), and CYP2E1 (E, F) in male (left) and female (right) offspring of saline and 100-DEX treatment groups. Data is expressed as mean fold change ± SEM. ANOVA: Statistical significance between saline and DEX-treated groups is shown by: * p < 0.05, ** p < 0.01, and *** p < 0.001, and **** p < 0.001.

To further comprehend the genetic alterations in prenatally DEX-exposed animals that could predispose them to hypertension and cardiovascular complications, we chose to analyze the expression of phenylalanine hydroxylase (PAH), a member of the solute carrier family of Na+/H exchangers SLC9A3, and the cytochrome P450 oxidases E class CYP2E1 genes (Fig 6) due to their role in catecholamine biosynthesis and regulation. These genes were analyzed only in the 100-DEX dosage group. When compared to saline, PAH expression was 3.59 fold higher in males, and 8.13 fold higher in females. On the other hand, while SLC9A3 expression was increased by 5.40 fold in males, its expression was reduced 2.17 fold in the females compared to the saline group. The expression of CYP2E1 in the males was decreased 4.16 fold, while in the females the expression of this gene remained unchanged compared to saline.

## Discussion

Previous studies have shown that prenatal exposure to GCs contributes to fetal programming of hypertension [25,29–31]. Increased PNMT expression, and its altered transcriptional regulation in brainstem adrenergic neurons, and in the adrenal gland have been proposed to be responsible for the overproduction of epinephrine and may conceivably be a genetic mechanism for the pathogenesis of hypertension [9,11,24,25].

### Fetal programming by DEX exposure is dose and sex dependent

Prenatal exposure to DEX in the third trimester is a paradigm for fetal programming of low birth weight offspring resulting in adulthood hypertension [7,25,26,30]. Low birth weight was observed in offspring prenatally exposed to DEX, and negatively correlated to the dose, where the highest dose resulted in the lowest birth weight compared to controls. These findings parallel a study on Sprague-Dawley rats prenatally DEX-exposed at concentrations of 25 and 50 μg/kg/day [32].

The lowest DEX showed higher weights compared to the saline group in both males and females possibly because at lower concentrations, DEX did not significantly inhibit fetal cellular growth and may be a consequence of excess adiposity. In non-human primates and in humans, low dose DEX resulted in heavier or equal weighted offspring, compared to controls [33]. Higher DEX doses however affected the birth weight more significantly, and catch-up growth trends were observed until the end of the study period, albeit at a lower rate. Individuals born with low birth weight may undergo a compensatory growth where either body weight is fully caught-up to the average at a young age, or grows slowly and never catches up to the average body weight, the latter may be the case in the present study [34].

Results from this study show that BP is positively correlated with the prenatal dose of DEX. Furthermore, males had significantly higher increases in BP compared to female offspring, a finding also reported in the literature [30,35]. Studies suggest that males are more vulnerable, while females are relatively protected from prenatal insults [36,37].

We have previously shown increases in transcripts of adrenal PNMT in male offspring of pregnant dams that received Dex 100 μg/Kg/day [25]. To the best of our knowledge, this is the first report showing a dose dependent response of prenatal exposure to DEX altering adrenal catecholaminergic gene expression, with sex-specific responses. While the males showed clear dose dependent increases in TH, DBH and PNMT, the females were resistant to alterations in these genes at the lower doses, suggesting a higher threshold for stress mediated alterations, and more resilience. Clinically, this finding is very relevant because it establishes that lower DEX doses administered during pregnancy will likely not have long-term consequences on CAs and BP in female offspring. Clinically, treatment with DEX remains a controversial issue due to its adverse side effects, and much more still needs to be considered such as duration of DEX injections, timing of injection during gestation, and also the sex of the offspring.

Published studies have demonstrated sex-specificity in the catecholamine biosynthesis, with cell-type specific effects of estrogen on the expression of TH and DBH, with dependency on estrogen receptor subtype [38]. Other researchers have shown that the Y chromosome linked SRY gene exerts multiple effects on male physiology, including effects on catecholamines, renin-angiotensin system, and blood pressure homeostasis [39,40]. Numerous studies suggest sexual dimorphism in the interaction of the HPA with the HPG (hypothalamic pituitary gonadal) axes, thus rendering females more prone to stress-related anxiety, depression and psychiatric disorders, while males are more prone to metabolic alterations [41]. These changes may arise from the differential activation and feedback inhibition of the HPA axis under situations of stress; numerous studies show sex based differences in CRF levels, activation of CRF response, ACTH levels and other factors such as functioning of GR [42,43]. Thus, inherent sex-specific differences in the sensitivity and functioning of the HPA may confer protection from stress mediated programing of hypertension in females. This is an area of research that needs further investigation to tease out sex-specific differences in CA biosynthesis and comprehend their role in the sex difference of HPA activation.

Stress-activated transcriptional regulators that modulate the PNMT promoter include Egr-1, AP-2, GR, and Sp1 [16,17,19,44]. Here, these transcription factors were increased in a dose and sex dependent manner. While, all 4 factors were elevated in the females at the highest dose, in the males, Egr-1 and GR were markedly increased while AP2 and Sp1 were only slightly altered even at the highest DEX dose, a finding comparable to our previous study on 100-DEX males [25]. Our data suggests that sex specific alterations in transcription factors might be crucial in the regulation of CA biosynthesis in prenatally stressed animals, and that Egr-1 may play a prominent role in PNMT regulation in adrenals of males given that it is significantly increased even at the lowest dose.

Our study showed no significant changes in Corticosterone; studies in the literature have shown variable results for prenatal stress mediated alterations in Corticosterone as this is confounded by the stressor, circadian rhythm changes associated with the stress paradigm, and age of the animals [45]. The estrus cycle also affects the levels in females. Epi levels also weren’t significantly altered in the prenatally stressed animals, with only trends toward increases seen in the males with the higher doses, as we have shown previously [25]. Despite this observation, the increases in PNMT and other catecholamines’ gene expression are relevant and important as one of the factors that can affect hypertension. Moreover, other studies have shown that the variability in baseline levels of these hormones is not uncommon, but rapid elevations in response to stress as well as behavioural changes observed in animals that are prenatally stressed by glucocorticoids are reflective of overall SA/HPA defects [46,47].

Adrenal PAH expression was significantly elevated in both males and females of 100-DEX offspring compared to the sex-matched saline group. Interestingly, of all the genes analyzed in our study, PAH showed significant baseline differences between males and females, with females expressing much lower PAH. Predominantly located in the liver, PAH biosynthesizes tyrosine from phenylalanine; tyrosine is further utilized for CA biosynthesis in the adrenal gland [48,49]. Thus PAH is critical in CA biosynthesis as it generates the precursor molecule that feeds into the pathway. The role of PAH in the adrenal gland, and in the stress response is just beginning to be discovered; it has been highlighted by Jacobson (2017) as a stress sensitive gene, and was elevated in adrenal glands of male rats exposed to chronic stress [50]. In a recent metabolomics study, serum from DEX-treated animals showed decreases in Phe and increases in Tyr, which were suggested as predictors for DEX-mediated effects on metabolism [51].

The SLC9A3 gene showed a more sex specific alteration in that males were more prominently affected with prenatal DEX, and showed marked elevations, while females showed a slight, but significant decrease. SLC9A3, a member of the solute carrier family, is a known Na+/H+ exchanger that functions in Na+ absorption in the intestine and kidney [52]. Studies show that disruption of genes in this family, and ensuing disturbances in electrolyte homeostasis can lead to alterations in BP [53]. Although studies in the adrenal expression of this gene are limited, SLC9A3 has been shown to be elevated in the adrenals of males in other models of stress sensitive hypertension [54].

Finally, cytochrome P450 enzymes are crucial in the metabolism and clearance of xenobiotics from the body. In our model, CYP2E was significantly reduced in the DEX-exposed males, however no significant changes were seen in the females. There are sex based and tissue based differences for the expression of CYP genes reported in the literature [55]. The endocrine system and sex hormones can complicate the regulation of CYPs, and our finding could be useful in understanding the differences in pharmacokinetic handling of drugs in prenatally stressed males versus females. Overall, the sex-specific effects seen in PAH, SLC9A3 and CYP2E1 genes likely play an important role in sex-specific responses to prenatal stress, and a comprehensive study needs to be undertaken to examine their role in the programming of hypertension.

In summary, this study provides possible molecular mechanisms elucidating increased production of PNMT mRNA in adrenal glands of prenatally stressed rats (Tables 1 and 2). Expression of catecholamine biosynthetic enzymes, PNMT transcriptional regulators and other stress sensitive genes was both dose and sex dependent in the offspring. Although, the low dose was sufficient to increase adrenal PNMT expression, it did not significantly increase BP in the offspring. Therefore, if feasible, a lower DEX dose may be a safer alternative in a clinical setting, although extrapolation to a corresponding dose in humans is difficult. The sex differences might be a mechanism by which males express higher PNMT than females, consequently resulting in the elevated BP of males. This research has implications for differences in sex-based physiology that would be of significance in evaluating the effects of prenatal stress on catecholaminergic systems, and to further comprehend predisposition to inheritance and epigenetic alterations in future generations.

**Table 1:** Summary revealing dose dependent differences in the expression of stress sensitive genes in male offspring.

MALES	10 DEX	50 DEX	100 DEX
**EGR-1**	**↑**	**↑**	**↑**
**GR**	−	−	**↑**
**SP1**	−	−	−
**AP2**	−	−	−
**TH**	**↑**	**↑**	**↑**
**DBH**	**↑**	**↑**	**↑**
**PNMT**	**↑**	**↑**	**↑**
**Blood Pressure**	**↑**	**↑**	**↑**

**Table 2:** Summary revealing dose dependent differences in the expression of stress sensitive genes in female offspring.

FEMALES	10 DEX	50 DEX	100 DEX
**EGR-1**	−	−	**↑**
**GR**	−	−	**↑**
**SP1**	−	−	**↑**
**AP2**	−	−	**↑**
**TH**	−	−	**↑**
**DBH**	−	−	**↑**
**PNMT**	**↑**	**↑**	**↑**
**Blood Pressure**	−	**↑**	**↑**
